# The effects of protein phosphatase inhibitors on the duration of central sensitization of rat dorsal horn neurons following injection of capsaicin

**DOI:** 10.1186/1744-8069-2-23

**Published:** 2006-07-17

**Authors:** Xuan Zhang, Jing Wu, Li Fang, William D Willis

**Affiliations:** 1 Department of Neuroscience and Cell Biology, The University of Texas Medical Branch, Galveston, TX 77555-1069, USA; 2 Division of Neurosurgery, Department of Surgery, The University of Texas Medical Branch, Galveston, TX 77555-0517, USA

## Abstract

Protein kinases and phosphatases catalyze opposing reactions of phosphorylation and dephosphorylation, which may modulate the function of crucial signaling proteins in central nervous system. This is an important mechanism in the regulation of intracellular signal transduction pathways in nociceptive neurons. To explore the role of protein phosphatase in central sensitization of spinal nociceptive neurons following peripheral noxious stimulation, using electrophysiological recording techniques, we investigated the role of two inhibitors of protein phosphatase type 2A (PP2A), fostriecin and okadaic acid (OA), on the responses of dorsal horn neurons to mechanical stimuli in anesthetized rats following intradermal injection of capsaicin. Central sensitization was initiated by injection of capsaicin into the plantar surface of the left paw. A microdialysis fiber was implanted in the spinal cord dorsal horn for perfusion of ACSF and inhibitors of PP2A, fostriecin and okadaic acid. We found that in ACSF pretreated animals, the responses to innocuous and noxious stimuli following capsaicin injection increased over a period of 15 min after injection and had mostly recovered by 60 min later. However, pre- or post-treatment with the phosphatase inhibitors, fostriecin or OA, significantly enhanced the effects of capsaicin injection by prolonging the responses to more than 3 hours. These results confirm that blockade of protein phosphatase activity may potentiate central sensitization of nociceptive transmission in the spinal cord following capsaicin injection and indicate that protein phosphatase type 2A may be involved in determining the duration of capsaicin-induced central sensitization.

## Background

Intradermal injection of capsaicin provides a useful and reversible experimental model for the study of an inflammatory pain state, characterized by hyperalgesia and allodynia [1-5]. Intradermal injection of capsaicin in human and primate subjects produces an acute inflammation, mechanical allodynia, primary heat and mechanical hyperalgesia, and secondary mechanical allodynia and hyperalgesia by activation of C- fibers and some Aδ fibers [3,4,6-8]. Capsaicin injection can cause changes in behavioral responses of rats to cutaneous stimuli and increase responses of nociceptive projection neurons in the dorsal horn of the spinal cord [8]. Presumably, changes in central processing of nociceptive information are responsible for the secondary mechanical hyperalgesia and allodynia that is induced by capsaicin [8-10].

It is believed that the prolonged time course of central sensitization depends on the activation of signal transduction cascades [8-10]. Central sensitization can be modulated, either up or down, by regulating the phosphorylation status of some crucial neuro-signaling proteins in the spinal cord. The opposing reactions of phosphorylation and dephosphorylation of proteins are catalyzed and balanced by protein kinases and protein phosphatases, respectively, and these proteins may have important effects in the control of intracellular events [11-22]. It is known that several protein kinases, such as PKC, PKA, PKG and CaMKII, affect the responses of dorsal horn neurons through phosphorylation of synaptic receptors and proteins involved in intracellular signal transduction pathways, and the consequences of this modulation can be central sensitization, long-lasting inhibition, and/or changes in gene expression [23-31]. However, the involvement of protein phosphatases (PP) in these events is less clear. Previous experiments in our laboratory suggest that PP2A, a serine/threonine specific protein phosphatase, plays an important role in nociceptive behavioral responses induced by intradermal injection of capsaicin [32].

This study was designed to assess the role of PP2A in the process of capsaicin-induced central sensitization. The effects of fostriecin (a specific PP2A inhibitor) and okadaic acid (a general inhibitor of both of PP1 and PP2A) on responses of nociceptive dorsal horn neurons were tested in rats following capsaicin injection. Okadaic acid methyl ester (OAME), a derivative of okadaic acid (OA) that lacks phosphatase inhibitory activity, making it suitable as a negative control for okadaic acid [33], was also used in the experiments. Some of the results have been reported in abstract form [34].

## Results

All of the neurons recorded in this study were classified as WDR cells and were in the lumbosacral enlargement of the spinal cord in the vicinity of a microdialysis fiber (within 750 μm) inserted across the dorsal horn [10,26,31]. The depth of the dorsal horn neurons ranged from 400 to 750 μm. Most of the cells were at depths around 600 μm, the level of laminae IV-VI in rats.

### Effect of capsaicin injection on activity of dorsal horn neurons

**Figure 1:**
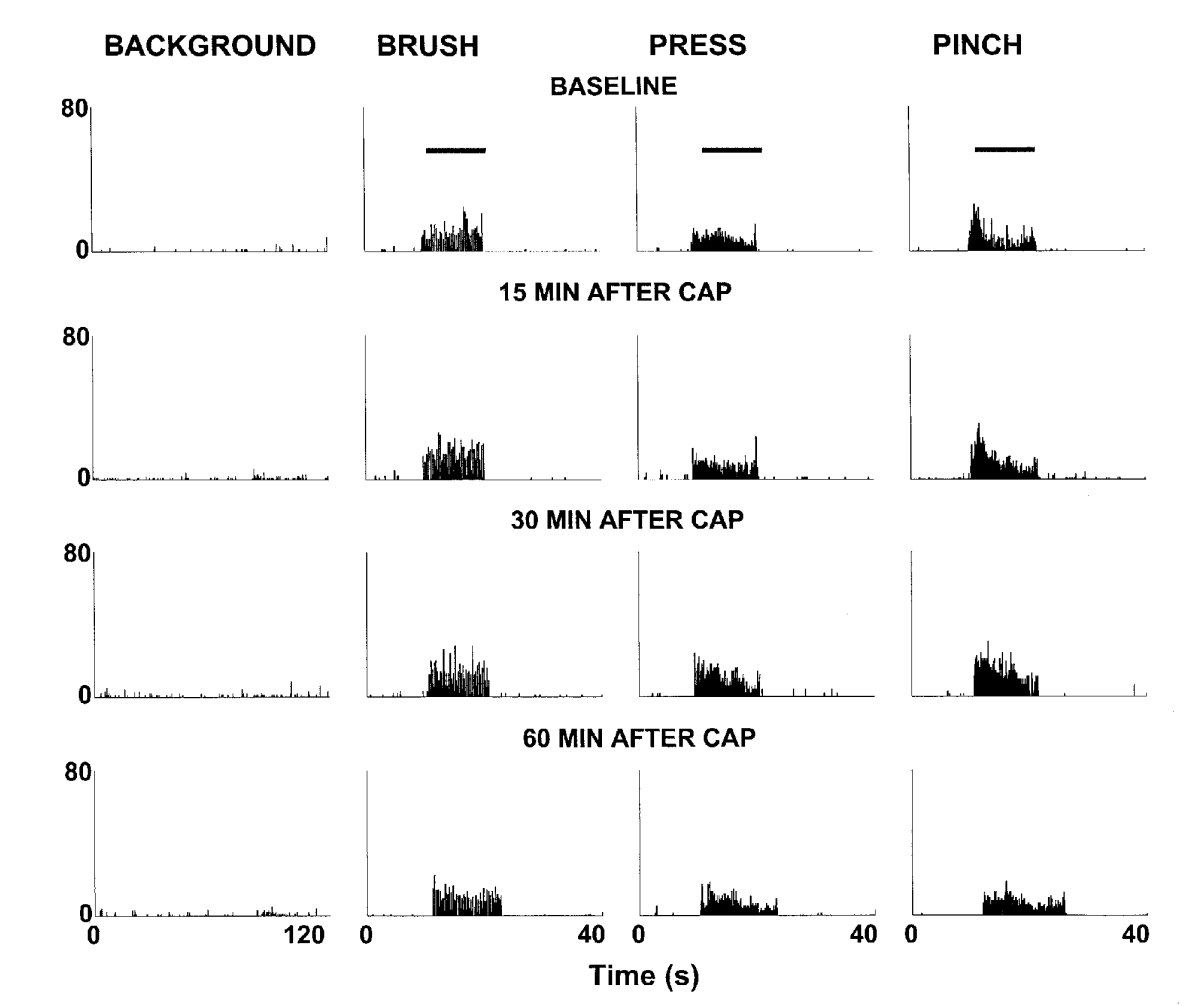
Effect of low dose capsaicin injection on the responses of a spinal dorsal horn neuron to mechanical stimuli. Rate histograms represent the responses to mechanical stimuli of a dorsal horn neuron before and following intradermal injection of capsaicin (0.1%, 10 μl). *Top row*: baseline background activity and responses to brush, press, and pinch; *Second row*: responses at 15 minutes after capsaicin injection. *Third row*: responses at 30 minutes after capsaicin injection. *Fourth row*: responses at 60 minutes after capsaicin injection.

**Figure 2:**
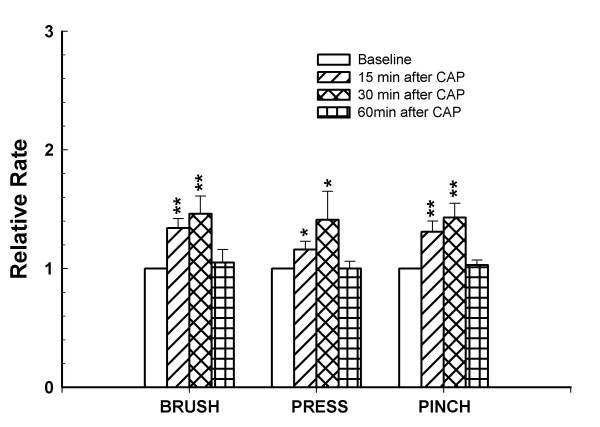
Bar graph shows the effects of intradermal injection of capsaicin on the responses of dorsal horn neurons to mechanical stimuli. All values are presented as mean ± SEM. *, *P *< 0.05, **, *P *< 0.01, *n *= 12.

The histograms in Fig. 1 show the responses of a representative dorsal horn neuron to graded mechanical stimulation of its receptive field and demonstrate the effects of the intradermal capsaicin injection. The top row shows the baseline background activity and the baseline responses to brush, press and pinch stimuli. After the baseline responses were recorded, capsaicin (0.1%, 10 μl) was injected into the plantar surface of the glabrous skin of the left hind paw of the rat. Significant increases were induced 15 minutes after the injection in background activity and in responses to brush, press and pinch stimuli (second row). The responses of the dorsal horn neuron reached a maximum level 30 min after the injection (third row) and had recovered from the effects of capsaicin by 1 h (bottom row). The grouped data from a total of twelve dorsal horn neurons are summarized in Fig. 2. These results demonstrate that intradermal injection of a low dose of capsaicin induces central sensitization of rat nociceptive spinal dorsal horn neurons and the duration of the central sensitization is less than 1 h for the dose of capsaicin used.

### Effect of PP2A inhibitors on responses of dorsal horn neurons

**Figure 3:**
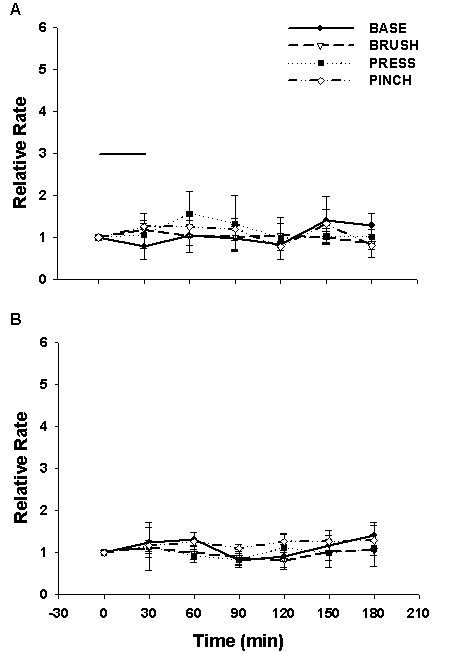
Effects of fostriecin (3 μM, n = 12, Panel A) or Okadaic acid (2 μM, n = 6, Panel B) infusion on the background activity and the responses of dorsal horn neurons to mechanical stimuli (BRUSH, PRESS, PINCH). Horizontal bar: drugs were continuously infused for 30 minutes.

To test if protein phosphatase 2A inhibitors, okadaic acid or fostriecin, will cause any change in dorsal horn neuron background activity and the responses to mechanical stimuli, the baseline responses of the dorsal horn neuron were recorded as a control. Okadaic acid (2 μM) or fostriecin (3 μM) was infused through the microdialysis fiber continually for 30 minutes. After the infusion, the background activity and responses to mechanical stimuli were recorded every thirty minutes until 3 hours following infusion. The grouped data of Fig 3 demonstrate that there were no significant effects of OA and fostriecin on the background activity or on the responses to the mechanical stimuli.

### Effect of a general inhibitor of PP2A and PP1, okadaic acid (OA), and its negative control (OAME) on the capsaicin-induced sensitization of dorsal horn neurons

**Figure 4:**
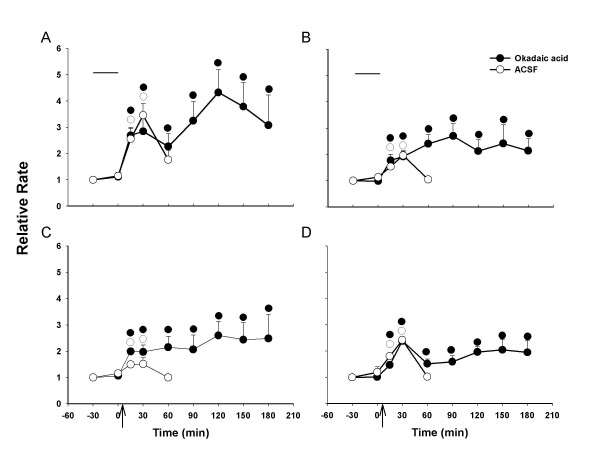
Effects of okadaic acid (OA, 2 μM) or ACSF infusion on the responses of dorsal horn neurons to mechanical stimuli following i.d. injection of capsaicin (arrows). OA (n = 6) or ACSF (*n *= 12) was continuously infused for 30 minutes before capsaicin injection (upper horizontal bars). The value before drug infusion was recorded as baseline. (A) Background activity; (B) Response to brush stimulus; (C) Response to press; (D) Response to pinch. ●○, P < 0.05, significant difference from baseline in each different lines. All values are presented as mean ± SEM.

**Figure 5:**
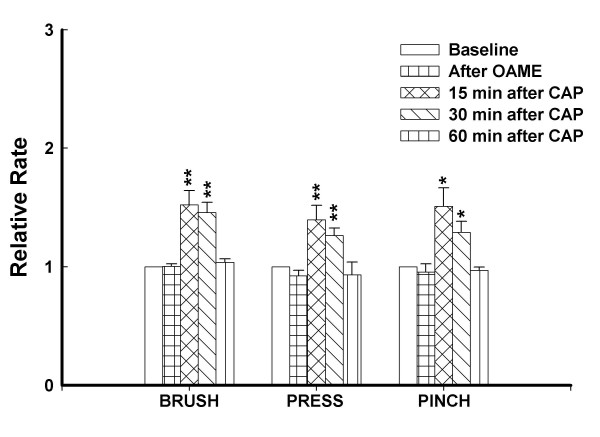
Bar graph shows the lack of effects of okadaic acid methyl ester (OAME, 2 μM) infusion on the responses of dorsal horn neurons to mechanical stimuli following capsaicin injection. OAME was continuously infused for 30 minutes before capsaicin injection. All values are presented as mean ± SEM. *, *P *< 0.05, **, *P *< 0.01, *n *= 8.

To test if the protein phosphatase activity is involved in the induction and maintenance of central sensitization, we studied the effects of okadaic acid (OA) and its negative control, okadaic acid methyl ester (OAME), on capsaicin-induced sensitization. After baseline activity of a dorsal horn neuron was recorded, 2 μM of OA or OAME was administered through the microdialysis fiber and the drug infusion was stopped after 30 min. There were no significant effects of OA and OAME on the background activity or on the responses to the mechanical stimuli. In the animals of the group that was treated with OA, capsaicin was injected intradermally and the responses were recorded every 30 min after capsaicin injection. In the animals of the group that was treated with OAME, the responses were recorded at 15 min, 30 min and 60 min after the injection. The grouped data from a total of six dorsal horn neurons are summarized in Fig. 4. The results suggest that the duration of central sensitization of rat nociceptive spinal dorsal horn neurons induced by intradermal injection of capsaicin is prolonged by pretreatment with OA. The increased responses did not recover by 3 hours following capsaicin injection, whereas in the OAME group, the time course of increased activity of nociceptive dorsal horn neurons was not impacted (Fig. 5).

### Effect of a specific PP2A inhibitor, fostriecin on the capsaicin-induced sensitization of dorsal horn neurons

**Figure 6:**
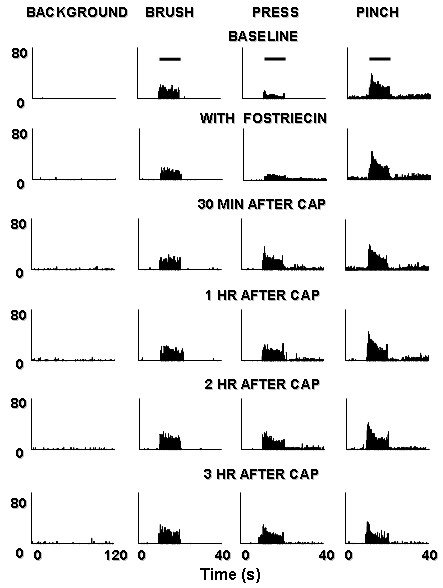
Rate histograms show the responses of a dorsal horn neuron to mechanical stimuli, the effect of fostriecin perfusion before capsaicin injection and the changes following intradermal injection of capsaicin. *Top row*: baseline background activity and responses to brush, press, and pinch. *Second row*: lack of effect of fostriecin infusion (3 μM) within the dorsal horn. The panels below indicate the effects of the injection of capsaicin after fostriecin infusion: at 30 min after capsaicin injection (*Third row*); at 1 h after capsaicin injection (*Fourth row*); at 2 h after capsaicin injection (*Fifth row*) and 3 h after capsaicin injection (*Bottom row*). Horizontal lines above responses in the top row indicate when the stimuli were applied.

**Figure 7:**
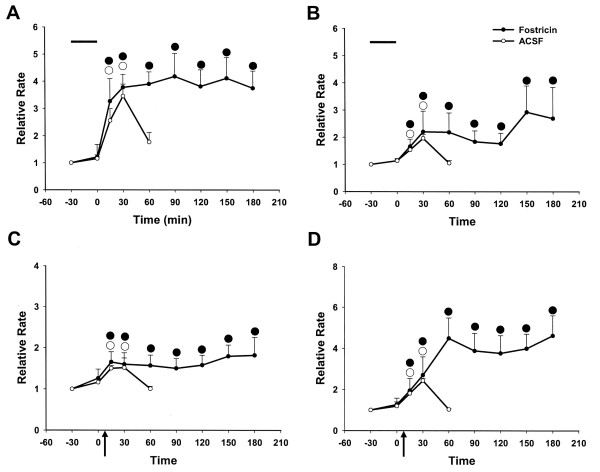
Effect of fostriecin infusion (3 μM; *n *= 10) or ACSF (*n *= 12) on increased activity of nociceptive dorsal horn neurons induced by capsaicin (0.1%, 10 μl) injection (arrows). Fostriecin or ACSF was continuously infused for 30 minutes before capsaicin injection (upper horizontal bars). The value before drug infusion was recorded as baseline. (A) Background acitivity; (B) Response to brush stimulus; (C) Response to press; (D) Response to pinch. ●○, P < 0.05, significant difference from baseline in each different lines. All values are presented as mean ± SEM.

The animals infused with fostriecin (3 μM) were divided into two groups. In one group, after the baseline activity of a dorsal horn neuron was recorded (Fig 6, top row), fostriecin was administered through the microdialysis fiber and the drug infusion was stopped after 30 min. There were no significant effects of fostriecin on the background activity or on the responses to the mechanical stimuli (second row). Capsaicin was injected intradermally and all responses were tested 15 minutes after the injection and repeated tests were done every 30 minutes until 3 hours after the injection. The rate histograms in Fig. 6 illustrate the effects of pretreatment with fostriecin in prolonging capsaicin-induced central sensitization. The grouped data from a total of ten dorsal horn neurons summarized in Fig. 7 show that after pretreatment with fostriecin, the increased activity of nociceptive dorsal horn neurons did not recover within 3 hours after capsaicin injection. By contrast, the increased activity induced by capsaicin in the dorsal horn neurons in the control group, which had an ACSF perfusion, had mostly recovered by 1 h. These results indicate that pretreatment with fostriecin can prolong the duration of central sensitization of rat nociceptive spinal dorsal horn neurons induced by intradermal injection of capsaicin.

**Figure 8:**
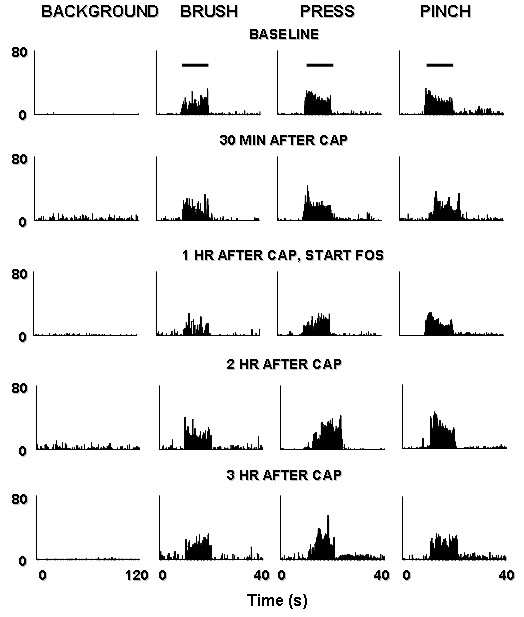
Rate histograms show the responses of a dorsal horn neuron to mechanical stimuli, the changes produced by intradermal injection of capsaicin and the effects of Fostriecin perfusion through the microdialysis fiber 1 h after i.d. injection of capsaicin. *Top row*: baseline background activity and responses to brush, press, and pinch. *Second row*: 30 min after capsaicin injection. *Third row*: 1 h after capsaicin injection. Fostriecin perfusion through the microdialysis fiber started. *Fourth row*: 2 h after capsaicin injection (Fostriecin perfusion stopped at 1.5 h after capsaicin injection). *Bottom row*: 3 h after capsaicin injection. Horizontal lines above responses in the top row indicate when the stimuli were applied.

In another group to test the post-treatment effect, after baseline activity of a dorsal horn neuron was recorded (Fig 8, top row), capsaicin was injected intradermally without pretreatment with fostriecin.

**Figure 9:**
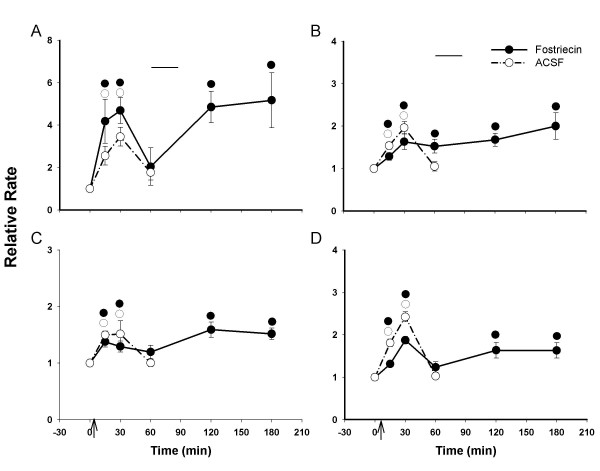
Effect of post-treatment with the phosphatase inhibitor fostriecin (3 μM; *n *= 12) or ACSF (*n *= 10) on increased activity of nociceptive dorsal horn neurons induced by capsaicin injection (arrows). 1 h after capsaicin injection, fostriecin or ACSF was continuously infused for 30 minutes (upper horizontal bars). The value before capsaicin injection was recorded as baseline. (A) Background activity; (B) Response to brush stimulus; (C) Response to press; (D) Response to pinch. ●○, P < 0.05, significant difference from baseline in each different line. All values are presented as mean ± SEM.

The infusion of fostriecin through the microdialysis fiber was started at one hour after capsaicin injection. The infusion was stopped after 30 min. The background activity and responses to brush, press, and pinch of the nociceptive neurons were recorded at 15 minutes after the injection and repeated every 30 minutes until 3 hours after the injection. The rate histograms in Fig 8 illustrate the effects of post-treatment with fostriecin on capsaicin-induced central sensitization. The grouped data from a total of twelve dorsal horn neurons summarized in Fig. 9 show that the increased activity of nociceptive dorsal horn neurons did not recover within 3 hours after capsaicin injection. Compared to the control group or ACSF treated group, these results suggest that post-treatment with fostriecin may prolong the duration of central sensitization of nociceptive spinal dorsal horn neurons induced by intradermal injection of capsaicin.

## Discussion

The role of serine/threonine protein phosphatase 2A in central sensitization during the peripheral inflammation induced by intradermal injection of capsaicin was investigated in this study.

In our previous behavioral study [32], we showed that a PP2A protein phosphatase inhibitor prolongs the time course of capsaicin-induced primary thermal hyperalgesia, secondary mechanical allodynia and hyperalgesia. In a preliminary electrophysiological study, we demonstrated that okadaic acid pretreatment may increase the duration of capsaicin-induced central sensitization of nociceptive dorsal horn neurons [34].

In the electrophysiology experiments, data were mostly recorded from single wide dynamical range (WDR) neurons in the spinal cord of anesthetized rats. In this case, intradermal injection of capsaicin induced central sensitization of nociceptive dorsal horn neurons of rats as shown by an increase in the background activity as well as in responses to both of non-noxious (brush) and noxious (press and pinch) mechanical stimuli. With ACSF infusion and a low dose of capsaicin, the capsaicin-induced central sensitization lasts about 1 hour. In our previous behavioral study, data were collected from awake rats [32], which are more sensitive to capsaicin injection compared to the anesthetized animals used in the electrophysiological experiments. On the other hand, in the current electrophysiological study, we detected the activity of single WDR neurons, while the nociceptive behavioral changes always result from the increased activity of many neurons. So the current results are consistent with the findings in our previous behavioral study.

Capsaicin-induced central sensitization was prolonged by administration of okadaic acid, a general inhibitor of PP2A and PP1, or fostriecin, a specific inhibitor of PP2A, through a microdialysis fiber. Based on previously published dose-response curves [32,35-37], the effective concentrations of these drugs are 30 nM for fostriecin and 20 nM for okadaic acid. To reach the calculated tissue doses for effective concentrations of fostriecin and okadaic acid, we applied 3 μM fostriecin and 2 μM okadaic acid in the dialysis fluid.

On the other hand, in normal rats, the activity and the responses to innocuous and noxious mechanical stimuli of nociceptive dorsal horn neurons were not modified by the PP2A inhibitor, and the time course of central sensitization was not prolonged by pretreatment with okadaic acid methyl ester, which was used as a negative control for okadaic acid. These observations strongly suggest that PP2A is involved in determining the duration of capsaicin-induced central sensitization.

Intracellular signal transduction pathways are critical regulators of a number of cellular events [12]. For nociceptive dorsal horn neurons, they play an important role in the responses to noxious stimuli. The opposing reactions of phosphorylation and dephosphorylation of participating proteins are crucial to such pathways. Protein kinases and phosphatases catalyze protein phosphorylation and dephosphorylation reactions, which may modulate the function of crucial proteins in the nervous system [11-20]. Serine/threonine specific phosphatases (PPs), which dephosphorylate serine and threonine residues in proteins, serve as one main component of protein phosphatases [22,38]. Serine/threonine phosphatases can be divided into PP1, PP2A, PP2B, PP2C, PP4, PP5, PP6 and PP7 [38]. Among them, PP2A is the most abundant phosphatase in mammalian cells and is expressed at high levels in the CNS [12,20,39]. In previous studies from our laboratory, we demonstrated that PP2A is highly expressed in the spinal cord of rats [40]. PP2A is also prominently involved in the regulation of specific signal transduction cascades, and it always exists in association with other phosphatases and kinases [41,42]. Activities of a number of kinases can be modulated by PP2A both *in vitro *and *in vivo*, particularly the ERK/MAPKs, the calmodulin-dependent kinases, PKA PKB, PKC, p70^S6 ^kinase, the I_κ_B kinases and the Cdks [42-44]. In rat adipocytes, PP2A is found to be the main phosphatase that regulates protein kinase B (PKB) [45]. Following an inflammatory stimulus, JNK, a kinase that participates in transcriptional activation, is regulated by PP2A [21]. The subcellular location and activity of PP2A can be dynamically modified in response to extracellular signals [46]. As an important regulator of signal transduction pathways in neurons, PP2A is also involved in spatial memory processes. Inhibition of PP2A results in the impairment of spatial memory retention by inducing Tau hyperphosphorylation [47].

As crucial neurotransmitters in the central nervous system, NMDA receptors act as nonselective cation channels important for neuronal excitability and particularly for Ca^2+^-dependent modulation of synaptic plasticity. PP2A was widely reported to participate in physiological long-term changes in an NMDA receptor dependent manner, such as long-term potentiation or long-term depression [20,38,48-50]. In the hippocampal CA1 area of rats, the depotentiation mediated by PP2A is induced by high-intensity theta-burst stimulation [51]. Since central sensitization of nociceptive transmission in the spinal cord can be viewed as a variety of long-term potentiation (LTP) [52,53], we hypothesize that PP2A may also modify the central sensitization in the spinal cord. This was supported by a study in mice, which indicated that inhibition of PP2A activity produced a dose-dependent counteraction of the antinociception induced by morphine [22].

The present unique research tested the role of a specific PP2A inhibitor, fostriecin, on evoked electrophysiological responses of nociceptive dorsal horn neurons in capsaicin injected rats. The present data demonstrate that fostriecin infusion into the spinal dorsal horn enhances the increased activity of nociceptive neurons induced by capsaicin injection by increasing the time course of the hyperexcitability. Based on previous studies in this laboratory, neither okadaic acid nor fostriecin by themselves altered the nociceptive behavior of rats [32]. This indicates that inhibition of PP2A does not affect the basal level of nociceptor activity and PP2A may not be involved in the initiation of central sensitization.

In a post-treatment paradigm, the infusion of the PP2A inhibitor one hour after capsaicin injection prolonged the central sensitization, which is normally reversed within one hour after a low dose of capsaicin injection. This result confirms that both the intracellular protein kinases and phosphatases are involved in the modulation of central sensitization. Intradermal injection of capsaicin induces the activation of both protein kinases and phosphatases [8,23,28,31,32,40]. Following the injection, the increased responses of WDR neurons gradually recovered and the effects of protein kinases and phosphatases are continually balanced during the process of central sensitization. While the phosphatase was inhibited by infusion of a PP2A inhibitor 1 h after capsaicin injection, the balance between phosphoryaltion and dephosphorylation was disturbed. The activity of WDR neurons increased again due to the prolonged central sensitization caused by the enhanced phosphorylation of critical proteins by persistent activation of protein kinases [8,31,40,53].

The microdialysis fiber technique that we used in this study may limit the drug effect to the dorsal horn, since a layer of silicon rubber was applied all along the length of the fiber except over a middle zone of 3 mm, which was used for drug dialysis into the dorsal horn of the spinal cord. Although we suspect that the drugs act post-synaptically, we could not rule out the possibility that the effects are on pre-synaptic nerve terminals [8,53].

The detailed mechanisms that may be involved in the present project could not be determined with our current experimental approaches. However, several possibilities may be considered. Since the activation of NMDA receptors is important for the initiation and maintenance of central sensitization following intradermal injection of capsaicin [53], the inhibitors of PP2A may prolong the duration of central sensitization by allowing a long lasting phosphorylation of NMDA receptors. It is known that several protein kinases, such as PKC, PKA, PKG, MAPK and CaMKII, affect the responses of dorsal horn neurons through phosphorylation of synaptic receptors and proteins involved in the intracellular signal transduction pathways, and the consequences of this modulation could be central sensitization, long-lasting inhibition, and/or changes in gene expression [23-31,53,54]. On the other hand, PP2A modulates the activities of a number of protein kinases, such as PKA, PKC and CaMKII [42-44]. So the inhibition of PP2A may prevent the dephosphorylation of protein kinases, and that may, in turn, affect the central sensitization.

## Methods

### Experimental animals

Male Sprague-Dawley rats weighing 250–300 g were used in this study. The protocols were approved by the Institutional Animal Care and Use Committee. These experimental protocols were also consistent with the ethical guidelines of the National Institutes of Health and of the International Association for the Study of Pain. All the animals used in the experiments were housed and maintained in accordance with the guidelines of the University of Texas Medical Branch (UTMB) Animal Care and Use Committee.

### Animal preparation

Rats used in the electrophysiology experiments were anesthetized with sodium pentobarbital (50 mg/kg). A laminectomy was performed to expose the spinal lumbar enlargement for electrophysiological recordings in dorsal horn cells. The spinal cord was exposed for a length of 3–4 cm and the rat was placed in a stereotaxic frame for recordings. A tracheotomy was carried out and a cannula was placed into the trachea for artificial respiration. End tidal CO_2 _was maintained at 4.0 ± 0.5%. A catheter was placed in the jugular vein for continuous administration of the anesthetic (5 mg/kg/h) and pancuronium (1 mg/kg/h) to paralyze the musculature. A controlled heating pad with feedback from the rectal thermometer probe was used to maintain the animal's body temperature at or near 37°C during the surgery and experiment.

### Microdialysis fiber construction

Cuprophan hollow fibers (150 μm inner diameter, 9 μm thick wall, 9 kDa molecular cutoff, from Spectrum Company) were used in the studies. A 30 cm-length Cuprophan fiber was marked with two 1 mm marks which were separated by a 3 mm space. Around the fiber on each side of the marks, a polyethylene sleeve (PE50 tubing) was placed. On each side of the marks, a few drops of silicon rubber were applied. The sleeve was moved back and forth to seal the membrane uniformly, including the two 1 mm marks. A thin layer of silicon rubber (Dow Chemical 3140 RTN coating) was applied all along the length of the fiber except over the middle 3 mm zone, which was to be used for dialysis. The fiber was left at room temperature overnight to dry. The dull end of a stainless steel dissecting pin (0.1 mm diameter, 0.5 mm in length) was inserted into the lumen of one end of the dialysis fiber. The pin was then glued in place in the lumen of the dialysis fiber with cyanoacrylate.

All drugs were administered through the microdialysis fiber, which was inserted into the spinal cord at a point approximately one-third the distance between the dorsal lateral sulcus and the dentate ligament and out through the other side at the same level. The 3 mm collection zone was placed across the dorsal horns bilaterally. The fiber end and pin were cut off and the fiber was glued to the end of a premeasured PE 20 tubing that was connected to a 5 ml syringe. The syringe was put on a Harvard infusion pump, which maintained a constant infusion rate of 5 μl/min [9,25].

### Administration of drugs

The drugs (concentration in the dialysis fluid given in parentheses) that were administered by microdialysis at 5 μl/min for about 30 min through the fiber are as follows: fostriecin, a potent serine/threonine protein phosphatase 2A inhibitor (3 μM, n = 10, pretreatment and 3 μM, n = 8, postreatment, Calbiochem, dissolved in ACSF), okadaic acid, a general inhibitor of both PP1 and PP2A (2 μM, n = 6), and okadaic acid methyl ester (OAME), which is suitable as a negative control for okadaic acid (2 μM n = 8; Calbiochem, dissolved in 10% DMSO and 90% ACSF). In previous articles from our lab, the assessment of the amount of diffusion across the microdialysis fiber in vitro of several similar-sized drugs with quite different chemical properties under ideal conditions was described [9,25,55]. Briefly, the applied drug was infused at the same flow rate and duration as that of the drug treatment used in the rats through a microdialysis fiber placed in a bath containing ACSF. The drug concentration was measured by a spectrophotometer (Beckman Coulter, Inc., Fullerton, CA) and the drug concentration diffusing across the fiber under ideal conditions was determined. In our experiment, the concentration ratio across the microdialysis fiber for all these drugs was around 1% and the drugs would be further diluted in the tissue before reaching their targets. Thus, the calculated tissue dose for effective concentrations for 3 μM fostriecin is 30 nM; the calculated tissue dose for 2 μM okadaic acid and OAME is 20 nM. These calculated tissue doses used throughout the results and figures in this article were supposed to be the effective concentrations of these drugs according to published dose response curves. [35-37]. Due to the drug's molecular weight, lipophilicity and tissue diffusion barriers, the actual tissue concentrations of the drugs may be less than the calculated concentrations in vitro. Therefore, the calculated dose is likely to be the maximal dose to which the tissue might be exposed.

### Injection of capsaicin

Capsaicin (0.1%, 10 μl) was injected into the plantar surface of the glabrous skin of the left paw of anesthetized rats. Capsaicin was dissolved in Tween 80 (7%), alcohol (20%) and saline. The dose of capsaicin was chosen to be low enough that it produced a central sensitization that lasted approximately 1 h in the absence of a phosphatase inhibitor.

### Electrophysiological recording

After the laminectomy, the animal was placed in a stereotaxic frame and held firmly with ear bars and a nose clamp. Vertebral clamps attached to the stereotaxic frame were used to hold the vertebral column stable. The nerve roots and spinal cord were seen after opening the dura over the exposed section of spinal cord. The spinal cord was covered and protected by warm mineral oil.

A carbon fiber microelectrode (0.4–0.8 MΩ, Kation Scientific) was used to make extracellular recordings of action potentials from dorsal horn neurons. The responses of dorsal horn cells to cutaneous brush, press, pinch and heat stimuli were evaluated. To record the timing and amplitude of action potentials and to analyze the data, the SPIKE2 computer software program and a multi-channel data-acquisition system, CED 1401Plus, were used.

The orthodromic responses of single cells to mechanical stimulation of a specific receptive field were recorded and dorsal horn neurons were identified based on their response patterns. At the beginning of the experiment for each neuron, the control background activity was determined first and then the activity evoked by mechanical stimuli was recorded periodically. BRUSH stimuli were applied by repeated brushing in a stereotyped manner with a camel's hair brush. PRESS and PINCH stimuli were delivered with arterial clips of different sizes, the pressures being 144 g/mm^2 ^and 583 g/mm^2 ^respectively. Usually, the background activity was recorded for 2 minutes before the application of mechanical stimuli. The stimulus began with an additional recording of background activity for 10 seconds, then the BRUSH, PRESS, or PINCH stimuli were delivered to the receptive field for 10 seconds. Each stimulus was applied to the same marked location, which was well removed from the injection site. Since the capsaicin would not reach and sensitize the nociceptor terminals at the stimulation site, increases in responses of dorsal horn neurons must be due to central, rather than peripheral, sensitization [27].

### Experimental design

The effect of the inhibitors of PP2A on the central sensitization of dorsal horn neurons following intradermal injection of capsaicin was tested in five groups of animals. In one group, capsaicin was injected following pretreatment with fostriecin infused through the microdialysis fiber for 30–40 minutes (3 μM), while in another group, fostriecin was infused 1 hour after capsaicin injection. The third group of animals was pretreated with okadaic acid (OA, 2 μM) and the fourth group was pretreated with okadaic acid methyl ester (OAME, 2 μM), through the microdialysis fiber as a negative control. In the control group, capsaicin was injected without any drug pretreatment. The background activity and responses to mechanical stimuli were recorded before the drug infusion for control purposes. To investigate the drug effect on dorsal horn neurons, all responses were recorded again after infusion of fostriecin, OA, OAME or ACSF. The responses were tested 5 and 15 minutes after the injection of capsaicin and repeated every 30 minutes until they had essentially recovered from the effects of the capsaicin injection.

### Data analysis

Recorded data were analyzed off-line from peristimulus time histograms processed by SPIKE2 software. Responses to mechanical stimuli applied to the receptive field for 10 sec were calculated by subtracting the background activity to yield a net increase in discharge rate. The relative rate was defined as response/baseline.

### Statistical analysis

The background activity and responses to mechanical stimuli of dorsal horn neurons were expressed as the mean ± SEM and analyzed by a Friedman repeated measures analysis of variance of ranks. For each combination, pair-wise multiple comparison procedures (Student-Newman-Keuls methods) were applied. P < 0.05 was accepted as significant. If overall significance was obtained, post-hoc testing was done using Dunnet's test to analyze the difference at different time points in the same group against the null hypothesis of no change. The difference between each group using an inhibitor and the control group at different time points after drug administration was analyzed by a post hoc Dunnet's test [9,25].
